# Anti-arthritic activity of aqueous-methanolic extract and various fractions of *Berberis orthobotrys* Bien ex Aitch

**DOI:** 10.1186/s12906-017-1879-9

**Published:** 2017-07-18

**Authors:** Alamgeer, Ambreen Malik Uttra, Umme Habiba Hasan

**Affiliations:** Laboratory of Cardiovascular Research and Integrative Pharmacology, Department of Pharmacology, Faculty of Pharmacy, University Of Sargodha, Sargodha, Pakistan

**Keywords:** Arthritis, Anti-oxidant, Berberidaceae, Phytochemical

## Abstract

**Background:**

The roots and stem bark of *Berberis orthobotrys* (Berberidaceae) have long been used traditionally to treat joint pain. Though, it has not been pharmacologically assessed for rheumatoid arthritis. The current study explores anti-arthritic activity and phytochemical analysis of aqueous-methanolic extract (30:70) and fractions (ethyl acetate, *n*-butanol, and aqueous) of *Berberis orthobotrys* roots.

**Methods:**

Anti-arthritic potential was evaluated in vitro using protein denaturation (bovine serum albumin and egg albumin) and membrane stabilization methods at 12.5–800 μg/ml concentration and in vivo via turpentine oil, formaldehyde and Complete Freund Adjuvant (CFA) models at 50, 100 and 150 mg/kg doses. Also, in vitro antioxidant ability was appraised by reducing power assay. Moreover, total flavonoid content, Fourier transform infrared spectroscopy and High performance liquid chromatography of *n*-butanol fraction were performed.

**Results:**

The results revealed concentration dependent inhibition of albumin denaturation and notable RBC membrane stabilization, with maximum results obtained at 800 μg/ml. Similarly, plant exhibited dose dependent anti-arthritic effect in turpentine oil and formaldehyde models, with maximum activity observed at 150 mg/kg. The results of CFA model depicted better protection against arthritic lesions and body weight alterations. Also, *B.orthobotrys* remarkably ameliorated altered hematological parameters, rheumatoid factor and positively modified radiographic and histopathological changes. Additionally, plant exhibited remarkable anti-oxidant activity. Moreover, phytochemical analysis revealed polyphenols and flavonoids.

**Conclusion:**

Taken together, these results support traditional use of *B.orthobotrys* as potent anti-arthritic agent that may be proposed for rheumatoid arthritis treatment.

## Background

Rheumatoid arthritis (RA) is an advancing, lingering, and a crippling disorder characterized by swelling, pain, and synovial joints stiffness. The exact etiology of this devastating disease is unknown. However, it is strongly linked to autoimmune reaction triggered by various genetic and external factors [1]. The normal use of available prevailing therapies constantly induce harmful consequences, which with the time may neutralize valuable outcomes. Plant drugs are favored over conventional medicines by patients because of unremitting quality of malady, fear of surgery, terrible morbidness, ever-growing medicinal cost, trivial reaction to established drugs and disadvantages of novel drugs. These herbal remedies diminish the manifestations of illness and raise the worth of life [2].

The genus *Berberis* (Berberidaceae) called “Zereshk” in Persian language comprises around 500 species. A few Berberis species i.e., *B. crataegina, B. aristata, B. vulgaris* and *B. calliobotrys* have been described to possess anti-arthritic activities [3–7]. *Berberis orthobotrys* Bien ex Aitch (Local name: Ishkeen) is an aromatic perennial shrub. It has been found in various parts of world. In Pakistan, it is widely distributed in Northern areas including Gilgit-Baltistan, where residents usually use roots and stem bark in powder/pills form for treating joint pain [8].

Preceding phytochemical studies have reported aporphine-benzylisoquinoline alkaloids namely berberine, berbamine, oxyacanthine, pakistanine, pakistanamine, kalashine and chitraline in *B. orthobotrys* [9]. Among these, berberine has been stated to have potential therapeutic implication in the treatment of RA due to its anti-proliferative effect against rheumatoid arthritis fibroblast like synoviocytes (RAFLS) [10]. Also, berberine has been reported to inhibit chronic inflammatory responses [6, 11–13]. In addition, berbamine exhibits immunosuppressive effect [14].

Hence, on account of traditional use of *B. orthobotrys* for joint pain, and the above mentioned effects of berberine and berbamine, the main constituents of *B. orthobotrys* against chronic inflammatory and immune responses, it was thought worthwhile to scientifically evaluate anti-arthritic activity of *B. orthobotrys* by means of both in vitro and vivo procedures, as no scientific data regarding anti-arthritic effect of this plant is existing to our utmost information.

## Methods

### Plant material

The roots of *Berberis orthobotrys* (Local name: Ishkeen) were collected from district Gilgit, Pakistan during the month of June, 2011 by Dr. Alamgeer, resident of village Shikiyote, District Gilgit. It was identified and authenticated by Dr. Shair Wali Khan, Assistant Professor Botany, Karakorum International University Gilgit Baltistan Pakistan. A voucher no. (BO-15-12) had been deposited in herbarium, Faculty of Pharmacy, University of Sargodha for future reference.

### Preparation of extract

The cold maceration process was used to prepare aqueous-methanolic (30:70) extract of *B.orthobotrys* root. The grounded plant material (2 kg) was soaked in 5 L of water-methanol mixture (30:70) for 72 h at room temperature with occasional stirring on daily basis. After three days, whole material was filtered through Whattman-1 filter paper and filtrate evaporated under reduced pressure on rotary evaporator. The crude extract was then air-dried to attain a solid mass, giving a yield of 18%. Afterwards, 100 g of plant extract was mixed with 500 ml of distilled water and partitioned with equal volume (500 ml) of ethyl acetate for three consecutive times. This provided 13 g of ethyl acetate fraction of *B. orthobotrys,* after collecting and evaporating ethyl acetate layer. The residual aqueous layer was further extracted with 500 ml of *n-*butanol for three consecutive times, thus providing 26.1 g of *B. orthobotrys n-*butanol fraction*,* after collecting and evaporating butanol layer. The remaining aqueous layer was evaporated and resulted in 60.9 g of aqueous fraction of *B. orthobotrys* [15]. The aqueous-methanolic plant extract and fractions were dissolved in distilled water for use in in vitro and vivo experiments.

### Chemicals

Bovine Serum Albumin (Sigma-Aldrich, USA), Fresh hen’s egg albumin, Complete Freund’s adjuvant (Sigma-Aldrich, USA), Formaldehyde (VWR, International Ltd), Turpentine oil (UNI-CHEM), Aspirin (UNI-CHEM), Diclofenac Sodium (Sigma-Aldrich, USA), Ascorbic acid (MERCK, Darmstadt, Germany). All the other chemicals used were of analytical grade.

### Animals

Sprague Dawley rats (either sex), weighing 200–300 g were purchased from University of Agriculture, Faisalabad, Pakistan. They were housed in stainless steel cages under controlled room temperature (25 ± 2 °C), with 12 h light/dark cycle and allowed *ad libitium* access to diet and water and received human care according to requirements of National Institute of Health (NIH) guidelines for care and use of laboratory animals. All the study protocols were approved by Institutional Animal Ethics Committee, Faculty of Pharmacy, University of Sargodha (Approval No. 20A25 IEC UOS). All the experiments performed complied with the rules of National Research Council [16].

### Pharmacological investigations

#### Evaluation of anti-arthritic effect of *Berberis orthobotrys* on inhibition of protein denaturation using bovine serum albumin (BSA)

The reaction mixture (0.5 ml) contained 0.45 ml BSA (5% aqueous solution) and 0.05 ml of different concentrations (12.5, 25, 50, 100, 200, 400, 800 μg/ml) of *B.orthobotrys* crude extract, fractions and aspirin (reference drug), correspondingly. Each solution was attuned to pH 6.3 by 1 N HCl. The samples were incubated at 37 °C for 20 min and heated at 57 °C for 30 min. Then phosphate buffer (2.5 ml) was added and absorbance was measured at 660 nm via spectrophotometer. For test control 0.05 ml distilled water was used instead of *B.orthobotrys* while product control lacked BSA [7]. The percentage inhibition of protein denaturation was deliberated by following formula:

$$ \mathrm{Percentage}\ \mathrm{inhibition}=100-\left[\frac{\mathrm{Abs}\ \mathrm{Test}\ \mathrm{Solution}-\mathrm{Abs}\ \mathrm{Product}\ \mathrm{Control}}{\mathrm{Abs}\ \mathrm{Test}\ \mathrm{Control}}\right]\times 100 $$

Abs = Absorbance

#### Evaluation of anti-arthritic effect of *Berberis orthobotrys* on inhibition of protein denaturation using egg albumin

The reaction mixture (5 ml) included egg albumin (0.2 ml), phosphate buffered saline, 2.8 ml (pH 6.4) and 2 ml of *B.orthobotrys* (crude extract and fractions respectively) and diclofenac sodium at various concentrations (12.5, 25, 50, 100, 200, 400 and 800 μg/ml), respectively. Equal volume of double-distilled water served as control. The mixtures were incubated at 37 ± 2 °C in a Biochemical oxygen demand (BOD) incubator for 15 min and then heated at 70 °C for 5 min. Their absorbance was measured at 660 nm [7]. The percentage inhibition of protein denaturation was appraised using undermentioned formula:

$$ \mathrm{Percentage}\ \mathrm{inhibition}=\left[\mathrm{Abs}\ \mathrm{control}\hbox{--} \mathrm{Abs}\ \mathrm{test}\ \mathrm{sample}/\mathrm{Abs}\ \mathrm{Test}\ \mathrm{Control}\right]\times 100 $$

Abs = Absorbance.

#### Evaluation of anti-arthritic effect of *Berberis orthobotrys* on human red blood cell (HRBC) membrane stabilization

The underlying method was used to perform membrane stabilizing activity [7]. Blood was taken from healthy human volunteers (informed consent was taken from blood donors to participate in the study, which was approved by Institutional Human Ethics Committee, Faculty of Pharmacy, University of Sargodha (Approval No. 12H09 IHEC UOS)), mixed with equivalent volume of sterilized Alsevers solution and centrifuged at 3000 rpm. The packed cells were washed with isosaline and 10% *v*/v suspension of red blood cells was prepared and used for study. Test solution consisted of 1 ml of phosphate buffer (pH 7.4, 0.15 M), hypotonic saline (2 ml), 0.5 ml of *B.orthobotrys* crude extract, fractions and diclofenac sodium at various concentrations (12.5, 25, 50, 100, 200, 400 and 800 μg/ml), respectively and 10% HRBC (0.5 ml). Test control solution comprised phosphate buffer (1 ml), distilled water (2 ml) and 10% HRBC (0.5 mL) in isotonic saline. Assay mixtures were incubated at 37 °C for 30 min, centrifuged at 3000 rpm, supernatant was decanted and hemoglobin content was estimated at 560 nm spectrophotometrically. The percentage membrane stabilization was projected using following formula:

$$ \mathrm{Percentage}\ \mathrm{protection}=100-\left[\left(\mathrm{Absorbance}\ \mathrm{sample}/\mathrm{Absorbance}\ \mathrm{control}\right)\times 100\right] $$

#### Evaluation of anti-arthritic activity of *B. orthobotrys* against turpentine oil induced joint edema in rats

The rats were fasted for 24 h prior to conducting experiment and were alienated into 14 groups (*n* = 5). Group I: Arthritic control rats (3 ml/kg distilled water). Group II: 100 mg/kg, p.o. aspirin. Group III, IV, V: *B.orthobotrys* crude extract at 50, 100, 150 mg/kg, p.o. respectively. Group VI, VII, VIII: *n-*butanol fraction (50, 100, 150 mg/kg, p.o. correspondingly). Group IX, X, XI: Ethyl acetate fraction at 50, 100, 150 mg/kg, p.o. accordingly. Group XII, XIII, XIV: Aqueous fraction (50, 100, 150 mg/kg, p.o. in that order). Inflammatory joint edema was developed by injecting 0.02 ml of turpentine oil into synovial cavity of right knee joint, 30 min following drug administration. Joint diameter was recorded with digital vernier calliper at hourly intervals for 6 h [7]. Percentage inhibition of knee joint edema by plant extract and aspirin was computed by correlating with untreated arthritic control rats, using following formula.

$$ \mathrm{Percentage}\ \mathrm{inhibition}=\frac{\mathrm{VC}-\mathrm{VT}}{\mathrm{VC}}\times 100 $$

VC = Joint edema of control group; VT = Joint edema of the test group.

#### Evaluation of anti-arthritic activity of *B. orthobotrys* against formaldehyde induced arthritis in rats

The rats were distributed into 14 groups (*n* = 5). Group I: Arthritic control rats (3 ml/kg distilled water). Group II: 100 mg/kg aspirin. Group III, IV, V: *B.orthobotrys* crude extract (50, 100, 150 mg/kg, p.o. respectively). Group VI, VII, VIII: *n-*butanol fraction (50, 100, 150 mg/kg, p.o. respectively). Group IX, X, XI: Ethyl acetate fraction (50, 100, 150 mg/kg, p.o. correspondingly). Group XII, XIII, XIV: Aqueous fraction (50, 100, 150 mg/kg, p.o. in that order). On day 1, 30 min subsequent to drug administration, arthritis was induced by sub plantar injection of 2% formaldehyde solution (0.1 ml) and recurrent induction on day 3. Drug treatment was sustained for 10 days. Arthritis was evaluated by checking mean increase in paw diameter for 10 days via digital vernier calliper [7]. Percentage inhibition of paw edema was worked out as described previously.

#### Evaluation of anti-arthritic activity of *B. orthobotrys* against complete Freund adjuvant (CFA) induced arthritis in rats

The rats were allocated into 7 groups (*n* = 5). Group I: Normal control rats (3 ml/kg distilled water). Group II: Arthritic control rats (3 ml/kg distilled water). Group III: Aspirin (100 mg/kg, p.o.). Group IV: *B. orthobotrys* crude extract (150 mg/kg, p.o.). Group V: *n-*butanol fraction (150 mg/kg, p.o.). Group VI: Ethyl acetate fraction (150 mg/kg, p.o.). Group VII: Aqueous fraction (150 mg/kg, p.o.). Arthritis was induced by injecting 0.05 ml of CFA subcutaneously into left foot pad of each rat. The treatments were administered to rats, 1 day ahead of CFA injection and daily treatment continued for 14 days. The injected paw edema was appraised at 1, 5, 10 and 15 days after CFA injection through digital vernier calliper. Percentage inhibition of edema was calculated as described earlier [7].

At 15th day, blood was collected by cardiac puncture for assay of hematologic parameters (RBC, WBC, Hb, ESR and Platelet count) and RF level. After that, legs of sacrificed rats were amputated at knee joints and radiographs of control and treated hind paws were taken with computerized radiographic system (Toshiba 630 MA, DS-TA-5A). Then histopathology of ankle joints was performed [7].

### Anti-oxidant activity

#### Evaluation of reducing power of *B. orthobotrys*

For the estimation of anti-oxidant activity, *B.orthobotrys* (extract and fractions, respectively) and standard ascorbic acid at various concentrations (12.5, 25, 50, 100, 200, 400 and 800 μg/ml) were mixed with 2.5 ml phosphate buffer (0.2 M, pH 6.6) and 2.5 ml potassium ferricyanide (1%). Mixture was incubated at 50 °C for 20 min. Then 2.5 ml TCA, trichloroacetic acid (10%) was added and centrifuged at 3000 rpm for 10 min. 2.5 ml of solution was mixed with 2.5 ml distilled water and 0.5 ml ferric chloride solution (0.1%). The absorbance was measured at 700 nm. A blank was prepared without adding extract [17]. Reducing power was calculated using below mentioned formula.

$$ \%\mathrm{increase}\ \mathrm{in}\ \mathrm{reducing}\ \mathrm{power}=\left[\mathrm{A}\ \mathrm{test}-\mathrm{A}\ \mathrm{blank}/\mathrm{A}\ \mathrm{blank}\right]\times 100 $$

A test is the absorbance of test solution; A blank is the absorbance of blank.

### Phytochemical investigations of *n-*butanol fraction

#### Total flavonoids content (TFC)

The total flavonoid content was found out using a previously described method with some modifications [18]. Briefly, *n-*butanol fraction (0.5 ml) was mixed with distilled water (2.2 ml) and 5% NaNO_2_ solution (0.15 ml). Then after 6 min, 10% AlCl_3_.6H_2_O solution (0.3 ml) was added and allowed to stand for 5 min. After that, 1 M NaOH (1 ml) was added and mixture was mixed by vortexing. Finally, absorbance was measured at 510 nm through spectrophotometer. Flavonols in *n-*butanol fraction were expressed as quercetin equivalent. Quercetin was used to perform calibration curve (standard solutions of 6.25, 12.5, 25.0, 50.0 and 100.0 μg/ml in 80% *V*/V ethanol).

### Fourier transformed infrared (FTIR) spectroscopic analysis

1 mg *n-*butanol fraction was taken in a mortar and pestle and grounded with 2.5 mg of dry KBr. The powder was then filled in a 2 mm internal diameter micro-cup and loaded onto FTIR set at 26 ± 1 °C. The samples were scanned using Fourier Transform Infrared Spectrometer (Thermo Nicolet Model-6700) in the range of 4000–400 cm^−1^ [19]. The spectral data obtained were compared with reference chart to identify functional groups present in the sample.

### High performance liquid chromatographic (HPLC) analysis

The sample preparation was attained by mixing *n-*butanol fraction (50 mg) with distilled water (16 ml) and methanol (24 ml). Thereafter, 6 M HCl (10 ml) was added. The mixture was kept for 2 h at 95 °C in oven, to get aglycons of flavonol glycosides. An HPLC analysis was performed using HPLC model LC-10A (Shimadzu, Japan), fitted with SCL-10A system control unit, Rheodyne injector, two LC-10 AT pumps, CTO-10A column oven, SPD-10A UV-Visible detector and data acquisition class LC-10 software. A filtered sample (20 μl) was injected into HPLC column. Two solvent systems A (H2O: Acetic acid ― 94:6, pH = 2.27) and B (Acetonitrile 100%) were used. Isocratic elution of mobile phase was used to perform chromatographic separation (mixture of solvent A and B (50:50 *v*/v), which was filtered using 0.45 μm membrane under vacuum before use at a flow rate of 1 ml/min at 30 °C. Detection was performed at a wavelength of 280 nm [20].

### Statistical analysis

The results were expressed as mean ± SEM and statistical analysis was carried out by one way ANOVA followed by Dunnett’s test and two way ANOVA followed by Bonferroni posttest using Graph Pad Prism 5.0 and *p* < 0.05 was considered statistically significant.

## Results

### Effect of *B.orthobotrys* on inhibition of protein denaturation using BSA

**Fig. 1:**
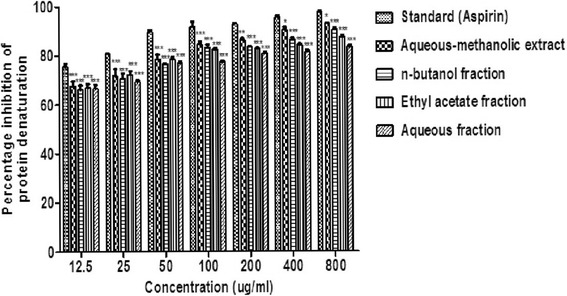
Effect of *B.orthobotrys* aqueous-methanolic extract and fractions against protein denaturation using bovine serum albumin. All the values are expressed as mean ± SEM (*n* = 3), using two way ANOVA followed by Bonferroni posttest. *** = *p* < 0.001, ** = *p* < 0.01, * = *p* < 0.05 vs aspirin

The inhibitory effects on protein denaturation are shown in Fig. 1. The present findings exhibited a concentration dependent impediment of protein denaturation by *B.orthobotrys* as well as aspirin throughout the concentration range (12.5–800 μg/ml). Crude extract demonstrated 92.81% inhibition of protein denaturation at 800 μg/ml, which was parallel to aspirin i.e., 97.55% at 800 μg/ml while, *n-*butanol fraction proved to be most efficacious among all fractions and its results were on a par with plant extract.

### Effect of *B.orthobotrys* on protein denaturation using fresh hen’s egg albumin

**Fig. 2:**
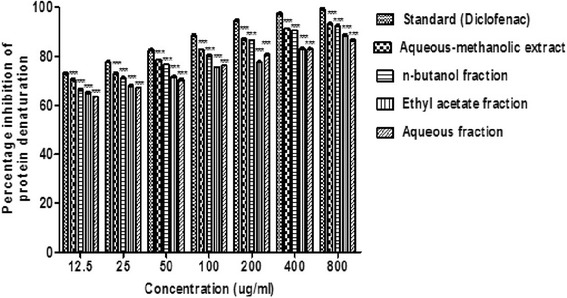
Effect of *B.orthobotrys* aqueous-methanolic extract and fractions on protein denaturation using egg albumin. All the values are expressed as mean ± SEM (*n* = 3), using two way ANOVA followed by Bonferroni posttest. *** = *p* < 0.001 vs diclofenac sodium

*B.orthobotrys* at several doses (12.5–800 μg/ml) provided considerable protection against denaturation of egg albumin. The results summed up in Fig. 2 point out that crude extract produced 93.30% inhibition of protein denaturation (*p* < 0.001) at 800 μg/ml. Among the fractions, *n-*butanol gave maximum results and showed 92.60% (*p* < 0.001) protection at 800 μg/ml whereas, diclofenac sodium brought about 99.19% suppression of protein denaturation at 800 μg/ml.

### Effect of *B.orthobotrys* on membrane stabilization

**Fig. 3:**
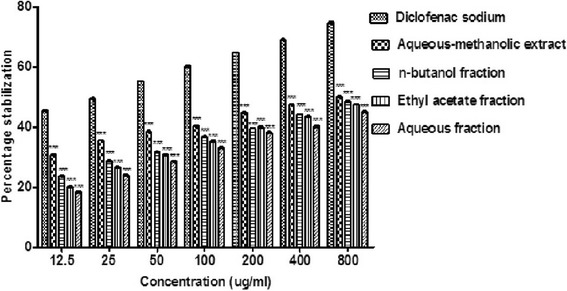
Effect of *B. orthobotrys* aqueous-methanolic extract and fractions on membrane stabilization. All the values are expressed as mean ± SEM (*n* = 3), using two way ANOVA followed by Bonferroni posttest. *** = *p* < 0.001 vs diclofenac sodium

In membrane stabilization model, there was a dose dependent increment in percentage protection (*p* < 0.001) for all concentrations (12.5–800 μg/ml) of crude extract and fractions albeit, it was much less than that of diclofenac sodium. The protective outcome detected with crude extract was 50.02% (*p* < 0.001) at 800 μg/ml however, *n-*butanol, most active fraction brought about 48.47% membrane stabilization at 800 μg/ml (*p* < 0.001). Diclofenac sodium exhibited maximum stabilization of 74.75% at 800 μg/ml (Fig. 3).

### Effect of *B.orthobotrys* against turpentine oil induced joint edema

The results given in Table 1 indicate that oral treatment with *B. orthobotrys* crude extract and fractions at 50, 100, 150 mg/kg exhibited highly significant (*p* < 0.001) concentration dependent suppression of synovial cavity swelling when compared to arthritic control. Following 6th h, percentage edema inhibition at 150 mg/kg plant extract turned out to be 73.75%, which was even more pronounced then that demoed by 100 mg/kg aspirin i.e., 71.27% (*p* < 0.001). Likewise, 150 mg/kg of *n-*butanol, ethyl acetate and aqueous fractions displayed 67.08%, 63.26% and 60.40% joint stability at 6th h respectively.

**Table 1:** Effect of *B.orthobotrys* on turpentine oil induced joint edema in rats (*n* = 5, Mean ± SEM)

Treatment groups	Increase in joint diameter (mm)					
	1 h	2 h	3 h	4 h	5 h	6 h
Arthritic control (3 ml/kg)	9.44 ± 0.028	10.59 ± 0.022	12.34 ± 0.023	14.98 ± 0.027	16.38 ± 0.028	20.96 ± 0.028
Standard Aspirin (100 mg/kg)	7.02 ± 0.026*** (25.63)	6.83 ± 0.026*** (35.50)	6.58 ± 0.035*** (46.67)	6.37 ± 0.024*** (57.34)	6.25 ± 0.028*** (61.84)	6.02 ± 0.027*** (71.27)
Aqueous-methanolic extract (50 mg/kg)	7.40 ± 0.021*** (21.61)	7.15 ± 0.021*** (32.48)	6.95 ± 0.030*** (43.67)	6.87 ± 0.026*** (54.13)	6.71 ± 0.026*** (59.03)	6.63 ± 0.024*** (68.36)
Aqueous-methanolic extract (100 mg/kg)	7.19 ± 0.021*** (23.83)	6.87 ± 0.027*** (35.12)	6.61 ± 0.022*** (46.43)	6.54 ± 0.021*** (56.34)	6.33 ± 0.026*** (61.35)	6.10 ± 0.024*** (70.89)
Aqueous-methanolic extract (150 mg/kg)	6.84 ± 0.026*** (27.54)	6.58 ± 0.025*** (37.86)	6.31 ± 0.024*** (48.86)	6.14 ± 0.022*** (59.01)	5.82 ± 0.023*** (64.46)	5.50 ± 0.025*** (73.75)
n-butanol fraction (50 mg/kg)	7.75 ± 0.018*** (17.90)	7.68 ± 0.019*** (27.47)	8.33 ± 0.024*** (32.49)	8.29 ± 0.029*** (44.65)	8.24 ± 0.029*** (49.69)	8.19 ± 0.027*** (60.92)
n-butanol fraction (100 mg/kg)	7.51 ± 0.023*** (20.44)	7.48 ± 0.023*** (29.36)	7.84 ± 0.021*** (36.46)	7.76 ± 0.027*** (48.19)	7.70 ± 0.025*** (52.99)	7.65 ± 0.019*** (63.50)
n-butanol fraction (150 mg/kg)	7.32 ± 0.021*** (22.45)	7.20 ± 0.021*** (32.01)	7.39 ± 0.021*** (40.11)	7.21 ± 0.021*** (51.86)	7.04 ± 0.028*** (57.02)	6.90 ± 0.023*** (67.08)
Ethyl acetate fraction (50 mg/kg)	7.96 ± 0.029*** (15.67)	7.86 ± 0.028*** (25.77)	8.44 ± 0.028*** (31.60)	8.41 ± 0.031*** (43.85)	8.05 ± 0.020*** (50.85)	9.10 ± 0.023*** (56.58)
Ethyl acetate fraction (100 mg/kg)	7.74 ± 0.021*** (18.00)	7.71 ± 0.026*** (27.19)	7.96 ± 0.027*** (35.49)	7.80 ± 0.028*** (47.93)	7.75 ± 0.025*** (52.68)	8.40 ± 0.021*** (59.92)
Ethyl acetate fraction (150 mg/kg)	7.43 ± 0.027*** (21.29)	7.40 ± 0.026*** (30.12)	7.73 ± 0.021*** (37.35)	7.48 ± 0.017*** (50.06)	7.22 ± 0.025*** (55.92)	7.70 ± 0.030*** (63.26)
Aqueous fraction (50 mg/kg)	8.24 ± 0.023*** (12.71)	8.11 ± 0.026*** (23.41)	8.60 ± 0.026*** (30.30)	8.92 ± 0.030*** (40.25)	9.13 ± 0.027*** (44.26)	9.58 ± 0.025*** (54.29)
Aqueous fraction (100 mg/kg)	7.98 ± 0.024*** (15.46)	7.77 ± 0.026*** (26.62)	8.10 ± 0.026*** (34.35)	8.45 ± 0.027*** (43.59)	7.78 ± 0.023*** (52.50)	8.99 ± 0.025*** (57.10)
Aqueous fraction (150 mg/kg)	7.62 ± 0.021*** (19.27)	7.54 ± 0.021*** (28.80)	7.69 ± 0.028*** (37.68)	7.95 ± 0.023*** (46.92)	7.23 ± 0.033*** (55.86)	8.30 ± 0.027*** (60.40)

Values in the parenthesis represent percentage inhibition of paw edema. The statistical analysis was carried out using two way ANOVA followed by Bonferroni posttest. *** = *p* < 0.001 when compared to arthritic control

### Effect of *B.orthobotrys* against formaldehyde induced arthritis

Results displayed in Table 2 depict that 150 mg/kg of crude extract and *n-*butanol fraction on 10th day showed more superior repression of paw edema i.e., 82.09% and 80.49% (*p* < 0.001) as compared to 78.54% reduction in paw edema by 100 mg/kg aspirin on 10th day. However, ethyl acetate fraction at 150 mg/kg showed appreciably comparable results to aspirin.

**Table 2:** Effect of *B.orthobotrys* on formaldehyde induced arthritis in rats (*n* = 5, Mean ± SEM)

Treatment groups	Increase in paw diameter (mm)				
	Day 2	Day 4	Day 6	Day 8	Day 10
Arthritic control (3 ml/kg)	8.49 ± 0.029	10.62 ± 0.023	12.56 ± 0.021	14.08 ± 0.020	16.92 ± 0.022
Standard Aspirin (100 mg/kg)	4.61 ± 0.021*** (45.70)	4.95 ± 0.023*** (53.38)	4.07 ± 0.019*** (67.59)	3.77 ± 0.028*** (73.22)	3.63 ± 0.033*** (78.54)
Aqueous-methanolic extract (50 mg/kg)	4.76 ± 0.035*** (43.93)	5.35 ± 0.026*** (49.62)	4.41 ± 0.018*** (64.88)	4.12 ± 0.021*** (70.73)	4.09 ± 0.019*** (75.82)
Aqueous-methanolic extract (100 mg/kg)	4.51 ± 0.015*** (46.87)	4.93 ± 0.023*** (53.57)	4.10 ± 0.028*** (67.35)	3.72 ± 0.029*** (73.57)	3.60 ± 0.027*** (78.72)
Aqueous-methanolic extract (150 mg/kg)	4.28 ± 0.011*** (49.58)	4.63 ± 0.016*** (56.40)	3.63 ± 0.030*** (71.09)	3.28 ± 0.023*** (76.70)	3.03 ± 0.017*** (82.09)
n-butanol fraction (50 mg/kg)	4.96 ± 0.019*** (41.57)	5.46 ± 0.020*** (48.58)	4.85 ± 0.025*** (61.38)	4.65 ± 0.024*** (66.97)	4.54 ± 0.027*** (73.16)
n-butanol fraction (100 mg/kg)	4.68 ± 0.023*** (44.87)	5.11 ± 0.021*** (51.88)	4.41 ± 0.021*** (64.88)	4.13 ± 0.021*** (70.66)	4.02 ± 0.021*** (76.24)
n-butanol fraction (150 mg/kg)	4.38 ± 0.026*** (48.40)	4.28 ± 0.020*** (59.69)	3.68 ± 0.023*** (70.70)	3.55 ± 0.021*** (74.78)	3.30 ± 0.023*** (80.49)
Ethyl acetate fraction (50 mg/kg)	5.40 ± 0.033*** (36.39)	5.70 ± 0.022*** (46.32)	5.32 ± 0.030*** (57.64)	5.00 ± 0.021*** (64.48)	4.91 ± 0.022*** (70.98)
Ethyl acetate fraction (100 mg/kg)	5.25 ± 0.021*** (38.16)	5.50 ± 0.021*** (48.21)	4.75 ± 0.017*** (62.18)	4.54 ± 0.022*** (67.75)	4.48 ± 0.022*** (73.52)
Ethyl acetate fraction (150 mg/kg)	4.75 ± 0.020*** (44.05)	5.05 ± 0.017*** (52.44)	4.37 ± 0.024*** (65.20)	4.00 ± 0.021*** (71.59)	3.85 ± 0.022*** (77.24)
Aqueous fraction (50 mg/kg)	5.88 ± 0.022*** (30.74)	6.13 ± 0.021*** (42.27)	5.71 ± 0.024*** (54.53)	5.37 ± 0.022*** (61.86)	5.25 ± 0.024*** (68.97)
Aqueous fraction (100 mg/kg)	5.43 ± 0.024*** (36.04)	5.96 ± 0.030*** (43.87)	5.18 ± 0.027*** (58.75)	4.85 ± 0.028*** (65.55)	4.75 ± 0.027*** (71.92)
Aqueous fraction (150 mg/kg)	4.98 ± 0.023*** (41.34)	5.58 ± 0.027*** (47.45)	4.47 ± 0.024*** (64.41)	4.25 ± 0.021*** (69.81)	4.20 ± 0.022*** (75.17)

Values in the parenthesis represent percentage inhibition of paw edema. The statistical analysis was carried out using two way ANOVA followed by Bonferroni posttest. *** = *P* < 0.001 when compared to arthritic control

### Effect of *B.orthobotrys* against CFA induced arthritis

**Fig. 4:**
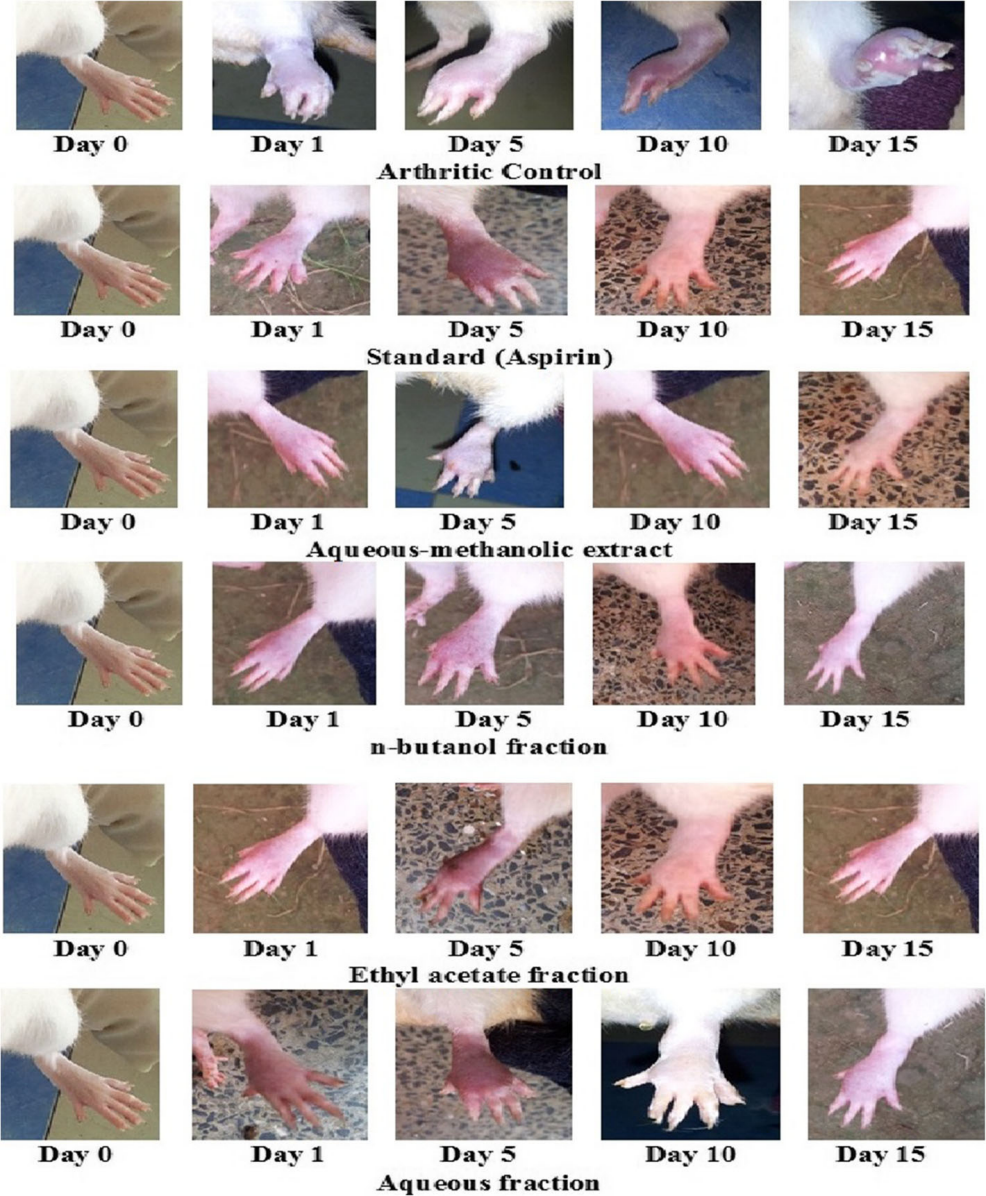
Photographic analysis (changes in paw diameter) of CFA induced arthritis in Sprague Dawley rats

**Fig. 5:**
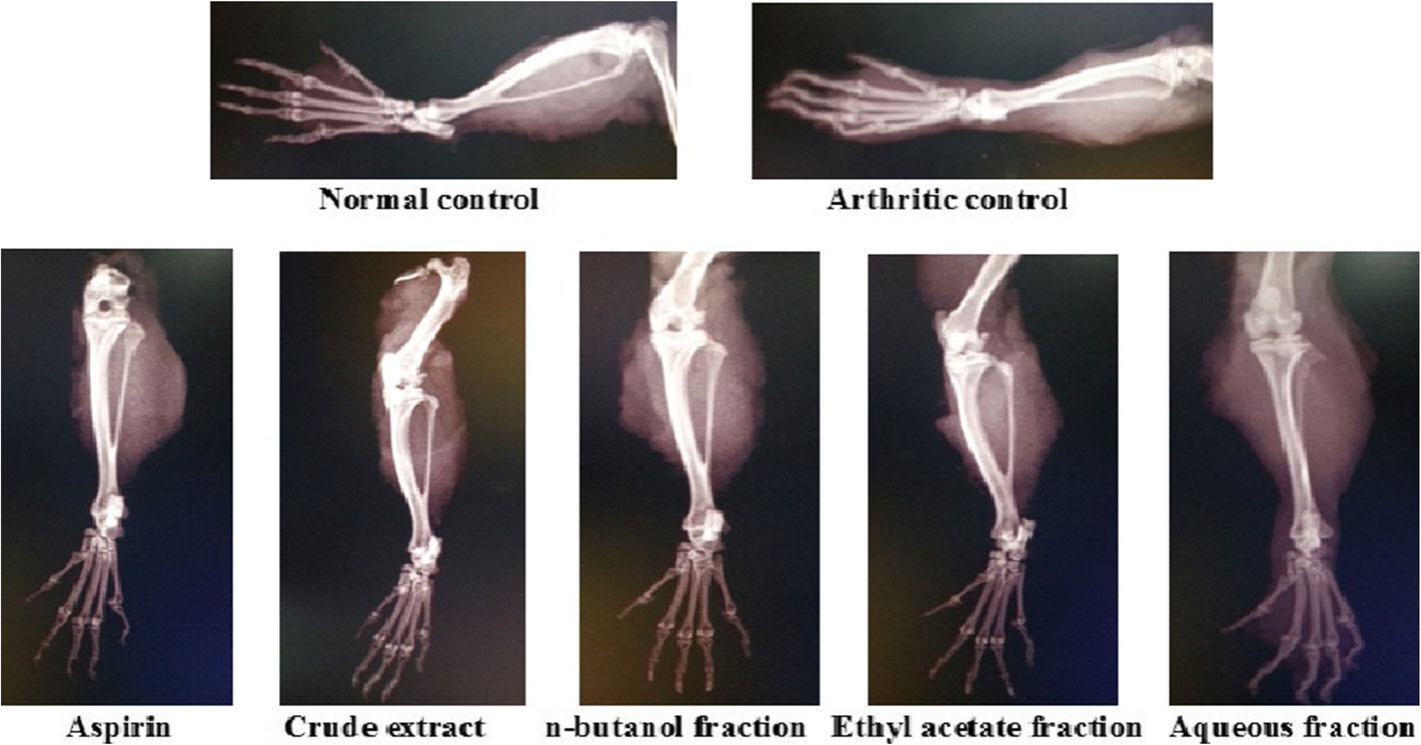
Radiographic analysis of CFA induced arthritis in Sprague Dawley rats at 15th day of treatment after CFA injection

**Fig. 6:**
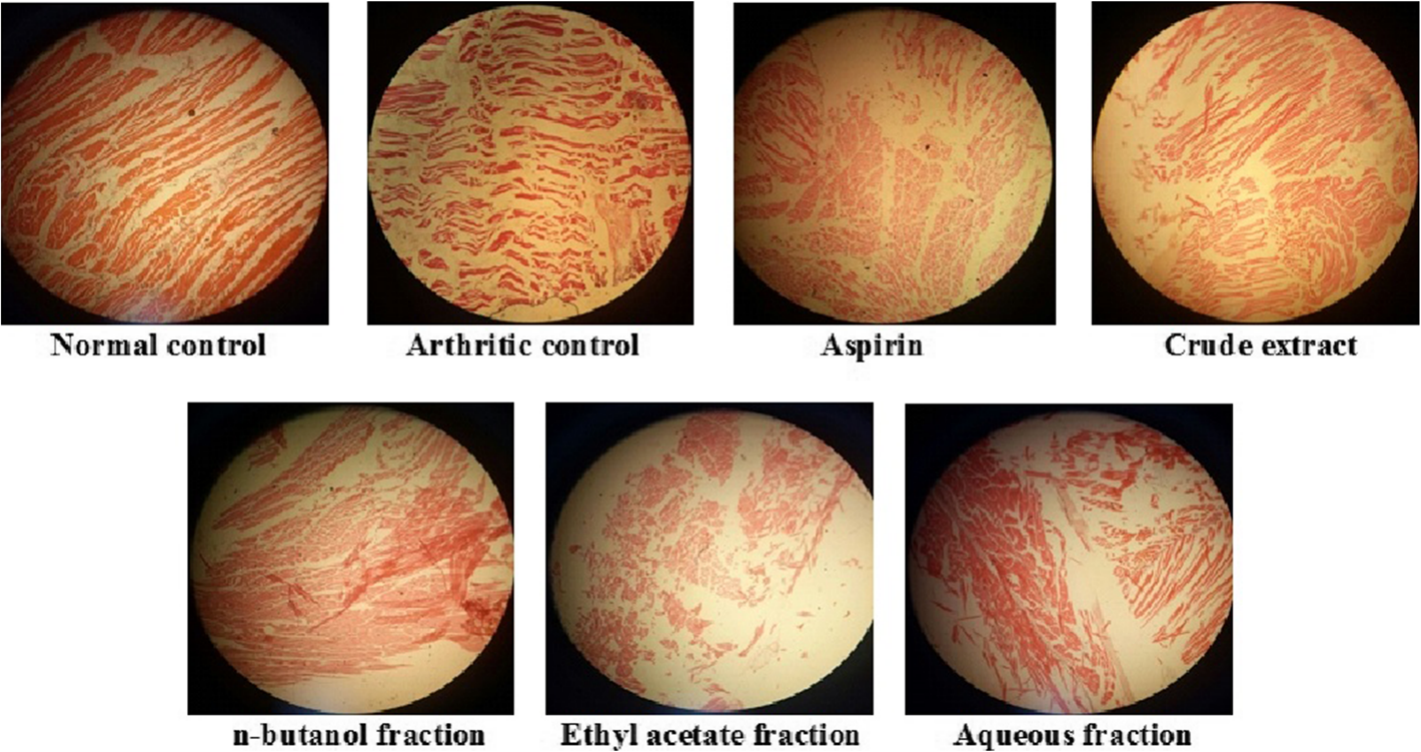
Histopathological analysis of ankle joint in Sprague Dawley rats at 15th day of treatment after CFA injection

Only the superlative dose (150 mg/kg) was selected for this method because anti-arthritic efficacy of plant extract has already been justified in aforementioned experiments. Table 3 shows a significant (*p* < 0.001) decrease in paw diameter of treatment groups as compared to CFA control group. Crude extract, *n-*butanol and ethyl acetate fractions at 150 mg/kg exhibited 80.98%, 78.24% and 76.60% inhibition of paw edema respectively at the end of study period. These results were more pronounced than 100 mg/kg aspirin i.e., 75.42%. Redness and swelling of affected joints were significantly less in treated as compared to arthritic control animals (Fig. 4). A significant (*p* < 0.001) weight gain was observed in treated groups at the end of study (Table 4). The CFA stimulated hematological disturbances and serum RF levels as depicted in Table 5, were positively altered by *B.orthobotrys* (extract and fractions) and aspirin. Figure 5 shows X-ray radiographs of left hind paws taken on 15th day. X-rays of normal control group showed no soft tissue swelling, normal bone and cartilage morphology. A limited abnormal pathology was revealed in radiographs of crude extract and *n*-butanol fraction treated rats. Whereas, intense swelling of soft tissues, stern joint destruction, discrete joint space reduction, marked bone resorption and bony erosions seen in arthritic control group were markedly reduced by aspirin and ethyl acetate fraction, however, aqueous fraction explicited mild protective effect. Histopathological changes in joints of arthritic rats are shown in Fig. 6. Histopathology of ankle joint of normal control rat expressed intact morphology of synovium and synovial lining, no inflammation and influx of inflammatory cells. The crude extract and *n-*butanol fraction presented remarkable inhibition of all histological findings of arthritis. The extremely abnormal histology of joint i.e., stern cartilage destruction, disturbed synovial lining, pronounced pannus formation, severe influx of inflammatory cells and erosive changes in cartilage and bone observed in arthritic control group was reversed moderately by aspirin and ethyl acetate fraction and mildly by aqueous fraction.

**Table 3:** Effect of *B.orthobotrys* on CFA induced arthritis in rats (*n* = 5, Mean ± SEM)

Treatments	Increase in paw diameter (mm)			
	Day 1	Day 5	Day 10	Day 15
Arthritic Control (3 ml/kg)	7.21 ± 0.035	16.22 ± 0.029	20.50 ± 0.024	23.72 ± 0.023
Normal Control	4.19 ± 0.160***	4.24 ± 0.166***	4.24 ± 0.097***	4.27 ± 0.079***
Standard Aspirin (100 mg/kg)	5.90 ± 0.034*** (18.16)	7.29 ± 0.030*** (55.05)	6.34 ± 0.029*** (69.07)	5.83 ± 0.029*** (75.42)
Aqueous-methanolic extract (150 mg/kg)	5.13 ± 0.029*** (28.84)	6.63 ± 0.029*** (59.12)	5.69 ± 0.027*** (72.24)	4.51 ± 0.033*** (80.98)
n-butanol fraction (150 mg/kg)	5.37 ± 0.030*** (25.52)	6.97 ± 0.033*** (57.02)	5.75 ± 0.029*** (71.95)	5.16 ± 0.032*** (78.24)
Ethyl acetate fraction (150 mg/kg)	5.51 ± 0.030*** (23.57)	7.68 ± 0.044*** (52.65)	6.47 ± 0.032*** (68.43)	5.55 ± 0.039*** (76.60)
Aqueous fraction (150 mg/kg)	5.82 ± 0.038*** (19.27)	8.22 ± 0.034*** (49.32)	6.95 ± 0.045*** (66.09)	6.02 ± 0.042*** (74.62)

Values in the parenthesis represent percentage inhibition of paw edema. The statistical analysis was carried out using two way ANOVA followed by Bonferroni posttest. *** = *p* < 0.001 compared to arthritic control

**Table 4:** Effect of *B. orthobotrys* on body weight of rats in CFA induced arthritis (*n* = 5, Mean ± SEM)

Treatments	Body weight gain (g)			
	Day 1	Day 5	Day 10	Day 15
Arthritic Control (3 mL/kg)	262 ± 3.647	223 ± 3.906	218 ± 3.555	208 ± 4.534
Normal Control	269 ± 6.038^ns^	270 ± 5.544***	270 ± 5.955***	274 ± 6.112***
Standard Aspirin (100 mg/kg)	243 ± 3.040**	246 ± 3.597***	243 ± 2.926***	245 ± 3.776***
Aqueous-methanolic extract (150 mg/kg)	265 ± 3.479^ns^	269 ± 4.176***	277 ± 4.179***	285 ± 4.320***
n-butanol fraction (150 mg/kg)	259 ± 4.841^ns^	260 ± 5.109***	261 ± 4.831***	263 ± 4.802***
Ethyl acetate fraction (150 mg/kg)	250 ± 2.922^ns^	251 ± 2.839***	252 ± 2.379***	253 ± 2.498***
Aqueous fraction (150 mg/kg)	242 ± 2.205**	242 ± 2.227**	243 ± 2.577***	246 ± 2.782***

The statistical analysis was carried out using two way ANOVA followed by Bonferroni posttest. *** = *p* < 0.001, ** = *p* < 0.01, ns = *p* > 0.05 compared to arthritic control

**Table 5:** Protective effect of *B.orthobotrys* on hematological profile in normal and experimental rats (*n* = 5, Mean ± SEM)

Hematological parameter	Arthritic control	Normal control	Standard (Aspirin)	Aqueous- methanolic extract	n-butanol fraction	Ethyl acetate fraction	Aqueous fraction
Hb (g/dl)	9.36 ± 0.145	14.26 ± 0.240***	12.60 ± 0.058***	13.40 ± 0.115***	13.36 ± 0.176***	12.63 ± 0.133***	11.70 ± 0.115***
RBCs (× 10^6^ / μl)	4.97 ± 0.079	7.42 ± 0.266***	6.72 ± 0.133***	6.80 ± 0.038***	6.53 ± 0.104***	6.30 ± 0.038***	5.78 ± 0.059**
WBCs (× 10^3^ / μl)	9.43 ± 0.203	5.23 ± 0.120***	7.70 ± 0.208***	6.20 ± 0.265***	6.80 ± 0.346***	7.50 ± 0.208***	8.50 ± 0.058*
Platelets (× 10^3^ / μl)	1225.00 ± 105.39	311.00 ± 3.215***	732.33 ± 4.631***	394.00 ± 3.512***	492.33 ± 3.528***	591.66 ± 2.728***	692.00 ± 3.786***
ESR (mm / 1st h)	20.33 ± 0.882	3.00 ± 0.577***	12.33 ± 1.202***	9.00 ± 0.577***	11.00 ± 0.577***	12.33 ± 0.882***	15.00 ± 0.577**
RF (IU / ml)	48.33 ± 2.028	14.00 ± 0.00***	25.33 ± 2.333***	20.00 ± 1.155***	25.66 ± 0.882***	32.66 ± 1.453***	35.33 ± 1.764***

The statistical analysis was done with one-way ANOVA followed by Dunnett’s multiple comparison test. *** = *p* < 0.001, ** = *p* < 0.01, * = *p* > 0.05 compared with arthritic control

### Reducing power of *B.orthobotrys*

**Fig. 7:**
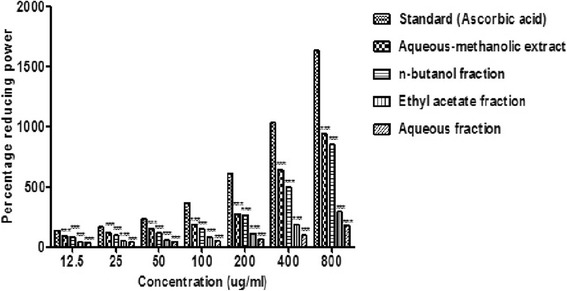
Effect of *B. orthobotrys* aqueous-methanolic extract and fractions on reducing power assay. All the values are expressed as mean ± SEM (*n* = 3), using two way ANOVA followed by Bonferroni posttest. *** = (*p* < 0.001) vs ascorbic acid

The outcomes of anti-oxidant screening described in Fig. 7 depict that *B.orthobotrys* crude extract displayed 944.59% (*p* < 0.001) anti-oxidant activity at 800 μg/ml. The reductive ability of ascorbic acid was found to be 1636.71% at 800 μg/ml. The most active fraction was found to be *n-*butanol which gave away highly significant (*p* < 0.001) anti-oxidant activity of 855.74% at 800 μg/ml and its efficacy was closer to crude extract.

### Total flavonoid contents in *n-*butanol fraction of *B.orthobotrys*

Total flavonoid content in *n-*butanol fraction was found to be 47.45 mg/g quercetin equivalent.

### FTIR analysis of *B.orthobotrys n-*butanol fraction

**Fig. 8:**
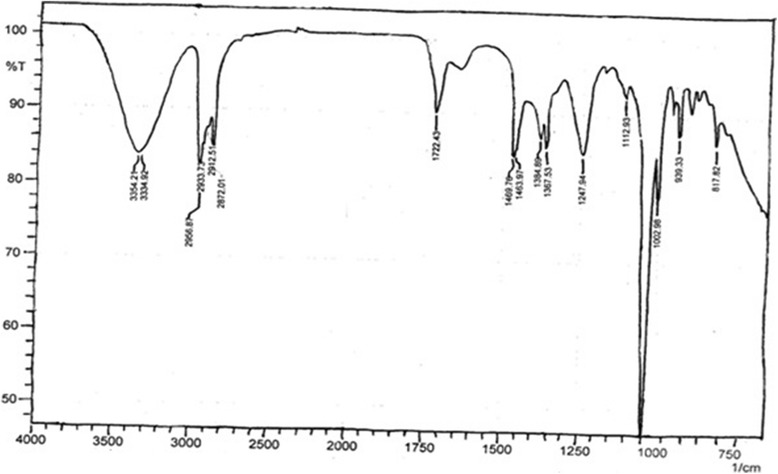
FTIR spectrum of *n*-butanol fraction of *B.orthobotrys*

Spectroscopic analysis was also performed for *n-*butanol, the most active fraction. A comparison of FTIR spectrum obtained with that of reference chart brought out the existence of phenols, alkanes, carboxylic acids, methyl, carbonyl and carbon fluorine functional groups in *n-*butanol fraction (Table 6 and Fig. 8).

**Table 6:** Functional groups identified by FTIR spectroscopy in *B.orthobotrys n*-butanol fraction

Peak	Bond	Functional group
3354.21 cm^−1^ - 3334.92 cm^−1^	OH Stretching vibrations	Phenols
2956.87 cm^−1^ – 2872.01 cm^−1^	CH Stretching vibrations alkanes	Alkanes
1722.43 cm^−1^	C = 0 carboxylic acid stretching vibrations	Carboxylic acids
1459.76 cm^−1^	CH_3_ bending vibrations	Methyl
1384.89 cm^−1^ – 1367.53 cm^−1^	C-O Stretching vibrations	Carbonyls
1037.7 cm^−1^	C-F stretching vibrations	Carbon fluorine

### Phenolic profile of *B.orthobotrys* n-butanol fraction

**Fig. 9:**
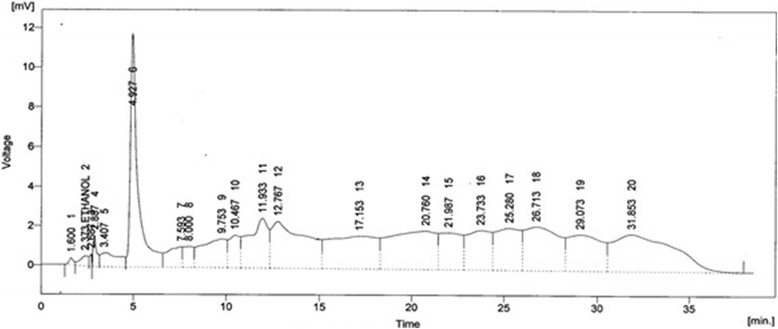
HPLC chromatogram of *n*-butanol fraction of *B.orthobotrys*

The most active fraction of *B.orthobotrys*, *n-*butanol was selected for phytochemical investigation. The HPLC analysis of *n-*butanol fraction revealed the presence of quercetin, gallic acid, caffeic acid, p-coumaric acid, M-coumaric acid, ferulic acid, trans-4-hydroxy-3-methoxy cinnamic acid and sinapic acid (Table 7 and Fig. 9).

**Table 7:** Phenolic constituents identified in *n*-butanol fraction of *B.orthobotrys*

Peak No.	RT (min)	Area Count	Amount (ppm)	Compounds
4	2.887	21.493	1.13	Quercetin
6	4.927	335.728	12.08	Gallic acid
12	12.767	305.143	14.03	Caffeic acid
13	17.153	297.613	3.86	p-coumaric acid
14	20.760	340.918	3.56	M-coumaric acid
15	21.987	152.704	10.94	Ferulic acid
17	25.280	197.301	4.53	Trans-4-hydroxy-3-methoxy cinnamic acid
18	26.713	278.642	3.61	Sinapic acid

## Discussion

The present study investigated in vitro and vivo anti-arthritic activity and in vitro anti-oxidant potential of aqueous-methanolic extract and fractions (*n-*butanol, ethyl acetate and aqueous) of *B.orthobotrys*. Moreover, phytochemical analysis was done using TFC, FTIR and HPLC. The major findings of this study included inhibition of protein (albumin) denaturation, HRBC membrane stabilization, inhibition of paw/joint edema and suppression of certain arthritic parameters by *B.orthobotrys*. Also, phytochemical studies depicted the presence of flavonoids and phenols.

In protein denaturation, secondary and tertiary structure of proteins is lost by extrinsic stress, heat, organic solvent or strong acid or base [21]. The mechanism of denaturation comprises variation in electrostatic, hydrogen, hydrophobic and disulphide bonding [22]. In the present investigation, plant extract got a higher inhibitory percentage of protein denaturation which was parallel to aspirin also, *n-*butanol fraction exhibited maximum repressing effect as compared to other fractions (Figs. 1 and 2). The increments in absorbance of test samples with respect to control indicated that *B. orthobotrys* has capability to bring down thermal denaturation of protein (albumin).

Since RBC membrane resembles lysosomal membrane so, effect of any agent on RBC stabilization may be projected to lysosomal membrane stabilization [23]. Moreover, strength of RBC’s rely on integrity of their membranes and contact of RBC’s to hypotonic medium leads to membrane lysis [24]. Also, impairment of lysosome membrane sets off the discharge of lysosomal constituents (bacterial enzymes and proteases) and phospholipase A_2_, inciting phospholipids hydrolysis to produce inflammatory intermediaries [7]. Thus, inhibition of RBC hemolysis in hypotonic media offers an additional mechanism of anti-arthritic effect. In the present study, plant extract and fractions presented an acceptable dose dependent stabilization of RBC membrane (Fig. 3). Membrane stabilizing attribute of *B.orthobotrys* could be allied with its interfering action on the release of neutrophils lysosomal content. Protective effect on erythrocyte lysis could possibly be acknowledged as an explicit indicator of anti-arthritic activity of *B.orthobotrys*.

Turpentine oil stimulated joint edema has been delineated by enhanced vascular penetrability and pronounced vasodilatation in which inflammatory intermediaries are discharged in 3 phases. The initiatory phase is thought to be mediated by serotonin and histamine, intermediate phase by kinin like substances and delayed phase by prostaglandins [25]. Previously aporphine-benzylisoquinoline alkaloids have been reported in *B.orthobotrys,* namely pakistanine, pakistanamine, kalashine and chitraline, in addition to berberine, berbamine and oxyacanthine [9]. In the present work, plant extract at 150 mg/kg dose exhibited more pronounced prevention of joint edema as compared to 100 mg/kg aspirin (Table 1). Impediment of edema intimates potential efficacy of *B.orthobotrys* on various phases of inflammation, which successively could be on account of presence of alkaloid berberine because formerly, it has also been proclaimed that berberine inhibits vascular permeability [6].

Inhibition of formaldehyde-induced edema is one of the most suitable methods to evaluate anti-proliferative activity and screen anti-arthritic agents. Formaldehyde develops localized inflammation in two phases. In early phase (neurogenic phase), substance P is discharged, while in late phase (inflammatory phase), histamine, serotonin, bradykinin and prostaglandins are liberated, which ensues in pronounced vasodilation and permeability [40]. It has been proclaimed that drugs that works on CNS inhibit both phases uniformly whilst, peripherally acting drugs inhibit late phase [7]. The results of present study showed that plant extract and *n-*butanol fraction at a dose of 150 mg/kg subdued proliferative edematous reaction greater than 100 mg/kg aspirin (Table 2), which tenably could be due to berberine, as *B.aristata* has previously been reported to inhibit formaldehyde induced arthritis owing to the presence of berberine [5]. Additionally, impediment of both early and late phases of formaldehyde response show that *B.orthobotrys* acts on CNS.

In the existing study, primary lesions instigated after 3–5 days of CFA injection while, secondary lesions took place after 11–12 days, featured by inflammation of non-injected hind paw. It has been reported that deformities in RA initiate due to the production of inflammatory mediators i.e., IFN α, PDGF and cytokines (IL-1, IL 6 and TNF-α) [26]. *B.orthobotrys* significantly inhibited arthritis in chronic stage and protected the joints as clear from histopathology results, which possibly could be due to its impeding action on inflammatory intermediaries as well as the result of immunological protection rendered by plant as supported by previous studies [13, 27]. RA is also allied with weight loss (rheumatoid cachexia) which may be ascribed to tissue destruction owing to proteolysis of muscle proteins by lysosomal proteases, mediated by PGE_2_ [7] and decreased absorption of ^14^C–glucose and ^14^C–leucine in rat’s intestine [28]. In the present work, evident restoration of rat’s body weight with 150 mg/kg of *B.orthobotrys* (Table 4) might be credited to reduction in PGE_2_ levels and increased absorption power of intestine. Additionally, this model gives a chance to analyze hematological alterations. The most frequent extracellular manifestation in RA occurring due to decrease in Hb and RBC levels is anemia, which ensues from decreased levels of erythropoietin [29] and reduced plasma iron, induced by IL-1 [7]. Moreover, liberation of IL-1 and TNF α in arthritic state causes rise in WBCs and platelet count [30]. Also, proteins (fibrinogen, α- and β- globulins) formed in reaction to inflammation increases ESR value [31]. Besides this, rheumatoid factor (RF) is a chief serologic marker in arthritis [7], which is an auto-antibody targeted against Fc segment of IgG. The results depicted that *B.orthobotrys* extract and fractions positively altered hematologic modifications (Table 5). As well, treatment with *B.orthobotrys* extract and fractions (most prominently *n-*butanol) presented noticeable decrease in radiographic and histological damage and markedly suppressed the modification in joint architecture (Figs. 5 and 6). It can be proposed that inhibition of CFA induced arthritis and its associated alterations might be accredited to the presence of alkaloid berberine and berbamine in *B.orthobotrys* as berberine has been described to show significant improvement in synovial hyperplasia and inflammatory infiltration via inhibition of TNF-α, IL-1β, IL-6, PGE2, COX-2, NF-kβ, repression of Th17, dendritic cell responses and other signaling pathways [11–13]. Formerly, an analysis carried out on *B.vulgaris* roots proved a marked repressive effect against CFA induced arthritis owing to the presence of berberine and oxyacanthine [6]. Moreover, berbamine exhibits immunosuppressive effect by stamping down STAT4 appearance and assembling of IFN-γ [14].

Reducing ability has been investigated from Fe^+3^ - Fe^+2^ transformation. Compounds possessing reducing capacity connote that they are electron donors, and have strength to quash the oxidized mediators that are produced as an upshot of lipid peroxidation mechanisms [32]. Extracts of various *Berberis* spp. (e.g. *B. aristata*, *B. vulgaris*, *B. croatica*, *B. microphylla* and *B. lycium*) have been described to hold anti-oxidative activities [32–36], which were proved to be associated with phenols and flavonols [34, 36]. Also berberine and berbamine, the main constituents of *Berberis* spp. have been detected to be anti-oxidants [37, 38]. Results of present study disclosed that *B.orthobotrys* owned preeminent puissance to knock off the free radicals (Fig. 7) that can be linked up with the presence of aporphine-benzylisoquinoline alkaloids [9] and reductones (phenols and flavonoids) in *B.orthobotrys*.

In present investigation as the active fraction was *n-*butanol so, its FTIR analysis revealed the presence of phenols, alkanes, carboxylic acids, methyl, carbonyl and carbon fluorine functional groups (Table 6 and Fig. 8). The presence of phenolics was further confirmed by HPLC and active principles isolated from *n-*butanol fraction were found to be quercetin, gallic acid, caffeic acid, p-coumaric acid, M-coumaric acid, ferulic acid, trans-4-hydroxy-3-methoxy cinnamic acid and sinapic acid (Table 7 and Fig. 9). It has formerly been reported that phenolics and flavonoids possess anti-inflammatory and anti-oxidant activities [39]. Previous works have shown that flavonoid quercetin exerts anti-inflammatory, anti-proliferative and anti-oxidative effects [40]. Similarly, gallic acid and phenolic acids i.e., p-coumaric, caffeic, and ferulic acids have been reported as anti-inflammatory and free radical scavengers [41, 42].

## Conclusion

In the view of above discussion, it is conceivable that *B.orthobotrys* Bien ex Aitch has been observed to exert significant anti-arthritic effect in experimental studies. Furthermore, *n*-butanol fraction has been avowed to be most potent. Though, exact mechanism of repressing the arthritic state by *B. orthobotrys* is not identified, its beneficial effects on RA could possibly be correlated with the presence of aporphine-benzylisoquinoline alkaloids (berberine, berbamine) detected previously in *B. orthobotrys* and phenols and flavonoids identified in the current study. In summary, this contemporary research lends pharmacological support to reported folkloric usage of *B.orthobotrys* in the treatment and management of painful arthritic inflammatory conditions. Based on our results, further thorough studies are required for appraisal of exact mechanism of action of *B.orthobotrys,* determination of pro-inflammatory cytokines level, isolation of active constituents and cellular characterization that could conclusively establish *B.orthobotrys* as a potentially safer disease modifying agent in the treatment of RA.

## Abbreviations

Abs: Absorbance
BSA: Bovine serum albumin
CFA: Complete freund adjuvant
ESR: Erythrocyte sedimentation rate
FTIR: Fourier transform infrared spectroscopy
Hb: Hemoglobin
HPLC: High performance liquid chromatography
HRBC: Human red blood cell
IFN α: Interferon-α
IL: Interleukin
PDGF: Platelet derived growth factor
PGE2: Prostaglandin E2
RA: Rheumatoid arthritis
RBC: Red blood cell
RF: Rheumatoid factor
TFC: Total flavonoid content
TNF-α: Tumor necrosis factor-α
WBC: White blood cell
